# Supplementary material for: The Impact of a Diet Containing Sucrose and Systematically Repeated Starvation on the Oxidative Status of the Uterus and Ovary of Rats
Source: Nutrients. 2019 Jul 8;11(7):1544. doi: 10.3390/nu11071544 (PMC6682934; doi:10.3390/nu11071544)
Supplement: Supplementary file 1 [file nutrients-11-01544-s001.pdf]

Table S1. Component composition of diets.

Component	Basic feed	Modified feed
Wheat (g/100 g)	36.4	6.01
Corn grain (g/100 g)	20.0	10.0
Wheat bran (g/100 g)	20.0	20.0
Dry whey (g/100 g)	3.0	3.0
Fodder salt[1] (g/100 g)	0.3	0.3
Soya-bean grain (g/100 g)	17.0	17.0
Fodder chalk[2] (g/100 g)	1.5	1.5
Phosphate 2-CA[3] (g/100 g)	0.8	0.8
Premix LRM[4] (g/100 g)	1.0	1.0
Wheat flour (g/100 g)	0.0	24.4
Sucrose (g/100 g)	0.0	16.0

[1] Mainly NaCl; [2]- Mainly $CaCO_3$; [3]- $CaHPO_4$; [4]- Vitamin-mineral composition used in animals feeds content per kg: IU: A 1500000, vit. D3, 100000; mg: vit. E 8000; vit. K 300, vit. B1 1200, vit. B2 1200, vit. B6 1000, vit. B12 8, Se 100, Fe 16000, Mn 4500, Zn 6000, Cu 1300, I 100, Co 200.

Table S2. Chemical composition of diets.

Component	Basic feed	Modified feed
Total protein (%)	17.9±0.09	16.9±0.10
% of diet energy	21.0	19.1
Crude fat (%)	3.25±0.11	3.56±0.09
% of diet energy	8.58	9.05
Carbohydrates (%)		
total	63.1±0.55	65.8±0.48
fiber	5.96±0.15	4.39±0.13
digested	57.1±0.50	61.5±0.44
% of diet energy	70.4	71.8
Dry matter (%)	90.8±0.20	92.9±0.11
Total ash (%)	6.52±0.12	6.63±0.18
Metabolizable energy		
(kcal/g)	3.41±0.03	3.54±0.04
(kJ/g)	14.2±0.21	14.8±0.25
Copper (mg/100 g)	1.8±0.09	1.4±0.08
Iron (mg/100 g)	20.1±0.71	15.8±0.068
Manganese (mg/100 g)	8.0±0.45	6.66±0.51
Selenium (mg/100 g)	0.037±0.001	0.033±0.001
Zinc (mg/100 g)	9.25±0.52	7.77±0.61

Table S3. Manufacturers and catalog numbers of reagents used in biochemical tests.

Assay	Manufacturer	Catalog number
CAT (red blood cell lysate)	Cayman Chemical	707002
CAT (supernatants)	Shanghai Sunred Biological Technology	201-11-5106
Estradiol	Fine Test Wuchan	ER1507
Glucose	BioSystems	11503
GPx (red blood cell lysate)	Cayman Chemical	703102
GPx (supernatants)	Shanghai Sunred Biological Technology	201-11-1705
Haemoglobin	Sigma Aldrich	MAK115
HDL-cholesterol	BioSystems	11557
Insulin	Demeditec Diagnostics	DE2048
LDL-cholesterol	BioSystems	11585
MDA (blood plasma)	Wuhan EIAab Sciences	E0597r
MDA (supernatants)	Shanghai Sunred Biological Technology	201-11-0157
Protein (supernatants)	Sigma Aldrich	B6916
SOD (red blood cell lysate)	Cayman Chemical	706002
SOD (supernatants)	Shanghai Sunred Biological Technology	201-11-0169
Total cholesterol	BioSystems	11805
Triglycerides	BioSystems	11828

CAT - catalase, GPx - glutathione peroxidase, MDA - malonyldialdehyde, SOD - superoxide dismutase
